# Inequalities in stunting among under-five children in Tanzania: decomposing the concentration indexes using demographic health surveys from 2004/5 to 2015/6

**DOI:** 10.1186/s12939-021-01389-3

**Published:** 2021-01-23

**Authors:** Edwin Musheiguza, Michael J. Mahande, Elias Malamala, Sia E. Msuya, Festo Charles, Rune Philemon, Melina Mgongo

**Affiliations:** 1 Department of Epidemiology and Biostatistics, Institute of Public Health, Kilimanjaro Christian Medical University College, P.O. Box 2240, Moshi, Tanzania; 2 Department of Mathematics and Statistics, College of Business Education, P.O. Box 1968, Dar es Salaam, Tanzania; 3 Department of Business Administration, College of Business Education, P.O. Box 1968, Dar es Salaam, Tanzania; 4 Data unit, Ifakara Health Institute (IHI), P.O. Box 78 373, Dar es Salaam, Tanzania

**Keywords:** Trend, Stunting, Inequality, Decomposition, Tanzania

## Abstract

**Background:**

Child stunting is a global health concern. Stunting leads to several consequences on child survival, growth, and development. The absolute level of stunting has been decreasing in Tanzania from from 50% in 1991/92 to 34% in 2016 although the prevalence is still high (34%)Stunting varyies across socioeconomic determinants with a larger burden among the socioeconomic disadvantaged group. The reduction of inequalities in stunting is very crucial as we aim to reduce stunting to 28% by 2021 and hence attain zero malnutrition by 2030 under Sustainable Development Goal 2.2.This study aimed at determining the trend, contributing factors and changes of inequalities in stunting among children aged 3–59 months from 2004 to 2016.

**Methods:**

Data were drawn from the Tanzania Demographic and Health Surveys. The concentration index (CIX) was used to quantify the magnitude of inequalities in stunting. The pooled Poisson regression model was used to determine the factors for stunting, decision criterion for significant determinants was at 5% level of significance. The CIX was decomposed using the Wagstaff and Watanabe decomposition methods., the percentage contribution of each factor to the toal concentration index was used to rank the factors for socioeconomic inequalities in stutning.

**Results:**

Inequalities in stunting were significantly concentrated among the poor; evidenced by CIX = − 0.019 (*p* < 0.001) in 2004, − 0.018 (p < 0.001) in 2010 and − 0.0096 (p < 0.001) in 2015. There was insignificant decline in inequalities in stunting; the difference in CIX from 2004 to 2010 was 0.0015 (*p* = 0.7658), from 2010 to 2015/6 was − 0.0081 (*p* = 0.1145). The overall change in CIX from 2004 to 2015/6 was 0.00965 (*p* = 0.0538). Disparities in the distribution of wealth index (mean contribution > 84.7%) and maternal years of schooling (mean contribution > 22.4%) had positive impacts on the levels of inequalities in stunting for all surveyed years. Rural-urban differences reduced inequalities in stunting although the contribution changed over time.

**Conclusion:**

Inequalities in stunting declined, differentials in wealth index and maternal education had increased contribution to the levels of inequalities in stunting. Reducing stunting among the disadvantaged groups requires initiatives which should be embarked on the distribution of social services including maternal and reproductive education among women of reproductive age, water and health infrastructures in remote areas.

## Background

Stunting is a global health concern caused by poor diet intake and recurrent infections [1]. Stunting leads to loss of physical growth potential and cognitive impairments in childhood [2]. During adulthood, stutning is positively associated with diabetes among women as compared to men [3]; higher levels of glucose and insulin and hence diminished function of beta cells which leads to increased insulin resistance [4]. A stunted child is at increased risk of cardiometabolic risk factors in adulthood including lower BMI, fat mass, systolic and diastolic blood pressure [5]. Furhtemore, stunting leads to morbidity and mortality [6]; increased risk of obesity due to impaired fat oxidation [7] as well as a 2–3 reduce years of school attendance, lower reading capacity, and 22% lower income in adulthood as compared to not stunted child [8].

Uneven distribution of stunting across socioeconomic determinants was termed as “socioeconomic inequalities in stunting” [9]. Socioeconomic inequalities in stunting are unjust as they result from the unfair distribution of resources, have spillover effects, and hence increases income inequalities [10]. Furthermore, inequalities in stunting may impact social gradient in health [11] and decelerated the achievement of Millennium Development Goals (MDGs) by 2015 [10].

Globally, stunting declined from 199.5 million in 2000 to 144.0 million children in 2019 [12] The prevalence of stunting varied across countries ranging from 2% in high-income countries to more than 50% in low-income countries [13]. Children living in the poorest wealth index had increased odds of being stunted by 1.2 to 1.7 folds in 2000 and 2014 respectively compared to a child living in the richest wealth index households [1]. Stunting declined in Asia from 136.6 million in 2000 to 78.2 million in 2019; in Latin America and Caribean from 9.5 million in 2000 to 4.7 million in 2019. A steady increase a steady increase from 49.7 million in 2000 to 57.5 million in 2019 was observed in Africa [12].

In Tanzania, stunting declined from 50% in 1991/92 to 34% in 2016; varying across socioeconomic determinants [14]. In 2015; about 39.9 and 19.2% lived in poor and rich wealth index; 37.8 and 24.7% lived in rural and urban areas while 39.3 and 26.1% were born to uneducated and secondary/higher educated mothers. Stunting raged from 15% in Dar es Salaam to 56% in Rukwa [14]. Studies done in Tanzania revealed that stunting is associated with socioeconomic and environmental factors [8, 15–19], although little is known basing on the magnitude and contributing factors of the evolvement of socioeconomic inequalities in stunting over time. Most of studies done in Tanzania [8, 15–19] used prevalence and classical regression techniques, our study aim to advance analysis by employing the Concentration Index (CIX) in quantifying the magnitude of socioeconomic inequalities in stunting.

Studies in developed and developing countries documented an increasing trend of socioeconomic inequalities in stunting. Socioeconomic inequalities were concentrated among the poor, mainly determined by disparities in household wealth index as well as maternal education [20, 21]. The difference between our studies to others is that we exluded children aged below three months as most of the stunting starts at three months [21]. This technique may help to minimize misclassification bias as clinicians argue that it is difficult to classify a newborn baby if is stunted or not.

Different interventions to mitigate stunting have been in place. The emphasis on exclusive breastfeeding for six months; continued breastfeeding, appropriate complementary feeding, provision of vitamin A supplementation, and deworming have been in place. Furthermore, the local production of nutritious food, provision of extra nutrients, and fortified foods as well as access to clean and adequate water and sanitation [14, 22, 23]. The nutrition-sensitive interventions and programmes in agriculture, use of social safety nets, early child development, and education [24] are vital towards the alleviation of malnutrition. Collective sectoral nutrition-sensitive approaches including women’s empowerment, agriculture, food systems, education, employment, social protection, and safety nets [25] are important in addressing child malnutrition.

Despite intervention in place, Tanzania observed a 0.8% annual reduction over 25 years. With this trend, is very unlikely to reach 28% by 2021 under Sustainable Development Goal (SDG) 2.2.1 and reduce inequalities in stunting thereby reaching zero malnutrition by 2030 under SDG 2.2. While one in three children was stunted in 2015, larger disparities in stunting across socioeconomic determinants persisted. This call upon the formulation of equity-based interventions which required evidence-based information. Therefore, this study aimed at determining the trend, contributing factors, and changes of socioeconomic inequalities in stunting among children aged 3–59 months from 2004/5 to 2015/6. Designing equity-based focused interventions might be a stepping stone in eradicating the observed socioeconomic inequalities in stunting and hence meet SDGs by 2030 thereby strengthening our national economy.

## Methods

### Study data and design

We analyzed the Tanzania Demographic and Health Surveys (TDHS) data using the most three recent surveys; TDHS 2004, TDHS 2010, and TDHS 2016. The methodology behind data collection of TDHS data has been described elsewhere [14, 20, 23]. The current study excluded children who had missing Height for Age Z- scores (HAZ). This study recruited only children who were living with their mothers in all surveyed year. This was because the survey year 2004 didn’t collect information on children not living with their mothers, hence consistence was needed for concrete trend analysis in all three suevey years.

Furthermore, because most of the stunting starts at 3 months [21], the current study recruited children aged 3–59 months. We included 7053, 6782, and 9215 children with complete information for surveyed years 2004, 2010, and 2016 respectively. The response rate was 6897 (98%), 6535 (96%), and 8258 (90%) for surveyed years 2004, 2010, and 2016 respectively. Data were accessed with the authorization of Demographic and Health Surveys (DHS). The study merged e Individual Recode (IR), Children’s Recode (CR) and the Household Member Recode (PR) datasets using unique identifiers. The HAZ score file for the surveyed year 2004 was independent of the child dataset hence further merging was done.

### Outcome and explanatory variables

The outcome variable was modeled in two different ways. Firstly, we used stunting as a binary variable (yes, no) when modeling the factors for stunting. Secondly, we used HAZ scores in their continuous form to model the factors for socioeconomic inequalities in stunting. Explanatory variables were grouped into four different categories including socio-demographic characteristics (age, sex, area of residence, the zone of residence, occupation, level of education), economic characteristics (household wealth index and land ownership for agricultural activities), maternal characteristics (early initiation of breastfeeding (breastfeeding was initiated within one hour after birth), use of Antenatal care (ANC), place of delivery, type of ANC and delivery attendant), Body Mass Index (BMI) for BMI (< 18.5 being underweight and ≥ 18.5 “not underweight”) and child characteristics (diarrhea status, age, sex) [6, 16, 18, 26–28]. The variables safe water and sanitation were not used as independent explanatory variables because are used in the construction of the wealth index [22].

The selection of explanatory variables to model socioeconomic inequalities in stunting was based on different literature [26, 27, 29–31]. Variables were treated basing on [32, 33] recommendations. Wealth index factor scores were used instead of wealth index, and the mother’s years of schooling was used instead of the mother’s level of education.

### A proxy measure of socioeconomic status

There are two proxy measures of socioeconomic status, household consumption and wealth asset index [32]. Because DHS does not collect information on household consumption levels, we used the wealth index. The wealth index is a composite measure constructed by the principal component analysis method. The computation of wealth index uses information on the household’s ownership of a number of consumer items (like telephone, radio, television, motorcycle, refrigerator, watch, mobile phone, bicycle, and car); dwelling characteristics (such as flooring material); type of drinking water source; toilet facilities; and animal ownership (like donkeys, buffalo, chickens, cattle, horses, goats, sheep, pigs). Each asset is assigned a weight (factor score) thereby standardizing them in relation to a standard normal distribution with a mean of zero and a standard deviation of one. Using these standardized scores, break points that define wealth quintiles are created. Five categories of quintiles namely the Lowest, Second, Middle, Fourth, and Highest are generated [34]. A detailed explanation of the computation may be found on [20, 22, 32].

### Measurement of inequalities in stunting

We used the concentration index (CIX) [32, 35] to quantify the unequal distribution of stunting across the wealth asset index. The concentration index is defined regarding the concentration curve as twice the area between the concentration curve and the line of equality. The concentration curve plots the cumulative percentage of the health variable (y-axis) against the cumulative percentage of the population, ranked by living standards, beginning with the poorest, and ending with the richest (x-axis) [32].

CIX was defined by the formula (eq. 1 below) where *μ* means the mean HAZ among children is aged 3–59 months, ℎ is the HAZ for each observation and *r* is the rank of the household socioeconomic status. The CIX takes values between − 1 and + 1; the value is negative when the burden of stunting lies among the poor, positive when the burden lies among the rich and zero when there is an equal prevalence of stunting across all socioeconomic groups [32, 33, 35].

### Decomposition of inequalities in stunting

The concentration indexes obtained for surveyed years 2004/5, 2010, and 2015/6 were decomposed to get the contribution of each determinant on the computed CIX. This decomposition was undertaken concerning linear regression models which links the continuous outcome variable with a set of determinants (eq. 2). For *β*_*k*_ being the coefficient of *x*_*k*_, *ɛ* being the error term (residual) while *y* is the dependent variable (HAZ) and ∝ is the constant term when all predictors equal to zero. Transforming eq. 2 to the CIX of stunting, the equation of decomposing the CIX may be written as in eq. 3. For *μ* being the mean of *y*, x̅_k_ is the mean of x̅k, *CIX*_k_ is the concentration index for x_k_ (the kth determinant) and *GC*_∈_ being the generalized concentration for error term (ε). The element (*β*_k_x̅_k_/*μ*) is an explained component while *GC*_∈_/*μ* is the unexplained component (residual).

For each explained component, there is elasticity *β*_k_x̅_k_/*μ* which indicates the impact of each *CIX* on the total *CIX* of the dependent variable *y*. We didn’t decompose the changes in the CIX of stunting due to insignificant changes in between the two consecutive surveyed years. If applicable, the total differential decomposition methods (eq. 4) of the changes in CIX could be applied to determine the contribution of each factor on the changes in CIX over years. This approach allows determining the impacts of the changes in the regression coefficients, the changes in the mean of the determinants of stunting, and the changes in the degree of inequality in the determinants of stunting.

1 $$ CIX=\frac{2}{\mu}\mathit{\operatorname{cov}}\kern0.5em \left(h,\kern0.5em r\right) $$

2 $$ y=\propto +\sum k\kern0.5em \beta k\kern0.5em \chi \mathrm{k}+\varepsilon $$

3 $$ \mathrm{CIX}=\sum \left({\upbeta}_{\mathrm{k}}{\overline{\mathrm{X}}}_{\mathrm{k}}/\upmu \right)\kern0.5em {\mathrm{CIX}}_{\mathrm{k}}+{\mathrm{GC}}_{\in }/\upmu $$

### Statistical methods

We used STATA version 14 in all analyses. Categorical data were summarized using frequency and percentages while continuous data were summarized using mean and standard deviation. The chi-square test was used to determine if there was a statistically significant difference between proportions. The CIX was computed using the index STATA command. A multivariable Poisson regression model was used to assess the association between stunting and explanatory variables. The study used the Poisson regression model instead of the logistic regression model because stunting had prevalence greater than 10% in each surveyed year hence termed as being a common outcome. The complex nature of the surveyed data was considered by applying the survey (svy) command. The statistical decision criterion was a 5% level of significance.

## Results

### Description of participant characteristics

Children were born to 4990, 4717, and 6273 mothers for the TDHS 2004/5, 2010, and 2015 respectively. During TDHS 2004/5; the majority were males 3385 (50%), born to mothers with primary education 1872 (26.1%), lived in rural areas 5621 (81%), and lived in the poorest wealth quintile households 1504 (22.8%). During TDHS 2010; the majority were males 3203 (49.7%), born to mothers with primary education 4051 (68.2%), lived in rural areas 5255 (80.5%), and lived in poorer wealth quintile households 1462 (23.8%). During TDHS 2015/6; the majority were males 4230 (50.6%), born to mothers with primary education 5068 (64.5%), lived in rural areas 6540 (74.2%), and lived in the poorest wealth quintile households 1931 (24.4%).

The mean child age was 29.7 (16.4), 29.2 (16.4), and 29.2 (16.4) months in the years 2004, 2010, and 2016 respectively. The average household size was 7 (4.2), 7.2 (3.8), 7.4 (4.2) for surveyed years 2004/5, 2010, and 2015/6 respectively. The overall mean HAZ was − 180.213 (134.4), − 168.6 (140.8), − 149.6 (135.2) in the years 2004, 2010, and 2015/6 respectively. (Table 1).

**Table 1:** Distribution of stunting among under-five children for TDHS 2004/5, 2010 and 2015/6

Characteristics	TDHS 2004/5 n (%)	TDHS 2010 n (%)	TDHS 2015/6 n (%)
**Area of residence**			
Urban	1159 (19)	1177 (19.5)	1903 (25.8)
Rural	5621 (81)	5255 (80.5)	6540 (74.2)
**Maternal education**			
No education	1872 (26.1)	1645 (25.6)	1855 (21.6)
Primary	4337 (69.4)	4051 (68.2)	5068 (64.4)
Secondary	571 (4.5)	736 (6.1)	1520 (14)
**Maternal BMI**			
< 18.5	270 (48)	313 (50.2)	250 (40.3)
≥ 18.5	2714 (45.2)	2326 (42.1)	2694 (35.3)
**Childbirth weight**			
Small	400 (40.2)	246 (51.8)	404 (48.9)
Average	2026 (46.8)	1844 (43)	2080 (35.4)
Large	553 (45.4)	439 (37.9)	438 (29.1)
**Wealth quintiles**			
Poorest	1504 (22.8)	1326 (21.7)	1931 (24.4)
Poorer	1360 (20.8)	1462 (23.8)	1770 (21.8)
Middle	1364 (21.5)	1378 (22.5)	1665 (19.5)
Richer	1501 (20)	1314 (18.6)	1736 (18.2)
Richest	1051 (14.9)	952 (13.4)	1341 (16)

TDHS represents Tanzania Demographic and Health Surveys

### Trends of child stunting by socioeconomic status

Over years, stunting declined from 45.5% in 2004 to 42.8 in 2010 and then to 35.6% in 2016. The prevalence of stunting significantly declined from 45.5% to 42.8% at p<0.001) for 2004/5 to 2010 respectively and then from 42.8% to 35.6% at p<0.001 for 2010 and 2015/6 respectively. The overall decline from 2004/5 to 2015/6 was also statistically significant at p<0.001 (all p-values computed according to Chi-square test for trend). Basing on socioeconomic status as measured by household wealth index, the larger decline was among the richer wealth quintile households while a small decline was among the richest wealth index. (

**Table 2:** Percentage of stunting among children aged 3–59 months by socioeconomic status

Survey year		Wealth quintiles			
	Poorest	Poorer	Middle	Richer	Richest
TDHS 2004/5	792 (52.3)	691 (50.2)	653 (48.8)	625 (46)	235 (23)
TDHS 2010	651 (49.3)	670 (46.1)	600 (45.5)	485 (39.5)	238 (26.6)
Difference (% point)	−3	−4.1	−3.3	−6.5	3.6
TDHS 2010	651 (49.3)	670 (46.1)	600 (45.5)	485 (39.5)	238 (26.6)
TDHS 2015/6	777 (41.5)	713 (40.6)	664 (40.2)	506 (30.2)	287 (20)
Difference (% point)	−7.8	−5.5	−5.3	−9.3	−6.6
TDHS 2004/5	792 (52.3)	691 (50.2)	653 (48.8)	625 (46)	235 (23)
TDHS 2015/6	777 (41.5)	713 (40.6)	664 (40.2)	506 (30.2)	287 (20)
Difference (% point)	−10.8	−9.6	−8.6	−15.8	−3

TDHS represents Tanzania Demographic and Health Surveys.

Table 2).

### Trends of socioeconomic inequalities in stunting

Over years, the levels of socioeconomic inequalities in stunting were significantly concentrated among the poor as the CIX had negative values in all surveys. The CIX was − 0.019 (*p* < 0.001), 0.0178 (p < 0.001) and − 0.0096 (p < 0.001) in 2004, 2010 and 2016 respectively. Socioeconomic inequalities in stunting insignificantly declined, the difference in CIX was 0.0015 (*p* = 0.7658) from 2004 to 2010 and 0.0081 (*p* = 0.1145) from 2010 to 2015. (Table 3).

**Table 3:** Trend of inequalities in stunting among children aged 3-59 months from 2004 to 2016

Year	CIX	***P***-value
TDHS 2004/5	− 0.0193	<0.001
TDHS 2009/10	− 0.0178	<0.001
**Difference**	0.0015	0.7658
TDHS 10	−0.0178	<0.001
TDHS 2015/16	−0.0096	0.0049
**Difference**	0.0081	0.1145
TDHS 2004/5	−0.0193	<0.001
TDHS 2015/6	−0.0096	0.0049
**Difference**	0.00965	0.0538

*CIX*: Concentration index.

TDHS represents Tanzania Demographic and Health Surveys.

### Socioeconomic determinants of the changes in child stunting

**Table 4:** The effect of socioeconomic factors on changes in stunting among children aged 3–59 months in the TDHS 2004/5, 2010 and 2015/6

Child stunting Characteristics	Phase I 2004/5–2010 APR (95% CI)	Phase II 2010–2015/6 APR (95% CI)	Phase III 2004/5–2015/6 APR (95% CI)
**Survey year**	0.96 (0 .9, 1.0)	0.8 (0.76, 0.88) ***	0.8 (0.74, 0.86) ***
**Area of residence**			
Urban	1	1	1
Rural	0.9 (0.8, 0.99)*	1.1 (0.93, 1.21)	0.97 (0.87, 1.08)
**Mother’s level of education**			
No education	1	1	1
Primary	0.99 (0.92, 1.06)	0.9 (0.85, 0.97)**	0.99 (0.93, 1.07)
Secondary	0.8 (`.64, 0.9) **	0.7 (0.63, 0.85) ***	0.9 (0.77, 1.01)
**Maternal BMI**			
< 18.5	1	1	1
≥ 18.5	0.89 (0.82, 0.99)*	0.89 (0.8, 0.97)*	0.96 (0.86, 1.1)
**Childbirth weight**			
Small	1	1	1
Average	0.85 (0.77, 0.9)***	0.72 (0.66,0.79)***	0.77 (0.7, 0.83)***
Large	0.72 (0.66, 0.79)***	0.62 (0.8, 1.1)***	0.63 (0.59, 0.69)***
**Wealth quintiles**			
Poorest	1	1	1
Poorer	0.9 (0.85, 1)*	0.9 (0.83, 0.99)*	0.9 (0.87, 1.01)
Middle	0.88 (0.81, 0.96)*	0.9 (0.82, 0.98)*	0.9 (0.84, 0.99)*
Richer	0.8 (0.76, 0.9) ***	0.8 (0.71, 0.87) ***	0.8 (0.74, 0.89) ***
Richest	0.5 (0.44, 0.6) ***	0.6 (0.48, 0.7) ***	0.5 (0.43, 0.59) ***

Adjusted for child age, child sex, child-size at birth; diarrhea status, maternal age, zones, cesarean delivery, marital status, skilled birth attendant, number of ANC visits, and year of interview

* Significant at *P* < 0.05; ** Significant at *P* < 0.01; *** Significant at *P* < 0.001

TDHS represents Tanzania Demographic and Health Surveys

Table 4 shows the results from the multivariable pooled Poisson regression models for the factors associated with stunting across two survey phases. We found that for phase I; children living in rural areas had 10% (APR=0.9, 95% CI: 0.81, 0.99) lower prevalence of getting stunted. Children born to mothers who had at least secondary education had 20% (APR=0.8, 95% CI: 0.64, 0.9), 30% (OR=0.7, 95% CI: 0.64, 0.86) lower prevalence of being stunted for phase I and II respectively. The prevalence of getting stunted decreased as the levels of household wealth increased; there were 50% (APR= 0.5 95% CI: 0.44, 0.6), 40% (APR= 0.6 (95% CI: 0.48, 0.7) and 50% (APR= 0.5 (95% CI: 0.43, 0.59) lower prevalence of getting stunted among children living in the richest wealth index households for phase I, II and III respectively. (Table 4).

### Decomposition of inequality in child stunting

Disparities inhousehold wealth index had a larger contribution on inequalities in stunting by increasing the CIX in all years although the contribution declined from 93% in 2004/5 to 72% in 2015/6. Differentials in maternal years of schooling was the second contributor of inequalities in stunting by increasing the levels of CIX from 9% in 2004/5 to 37% in 2015/6. Area of residence reduced inequalities in stunting in all surveys although the contribution changed over time by increasing then decreasing. The declined trend of the contribution of the household wealth index to the CIX is consistent with the declining inequalities in stunting measured by the CIX. While CIX declined over time, the contribution of mother’s years of schooling on the CIX increased over time. The insignificant decline of the CIX may be explained by the competing effect of household wealth and maternal years of schooling. The variables in this model explained inequalities in stunting more than 80%, the contribution of other factors not explained by the model varied over time as depicted by the residual. (Table 5).

**Table 5:** Decomposition of concentration indices for under-five stunting in the TDHS 2004/5, 2010 and 2015/6

Variables	**Stunting (height for age < 2 SD)**						
	**TDHS 2004/5**		**TDHS 2010**			**TDHS 2015/6**	
	CIX %		CIX %			CIX %	
Residence area	−0.07	−2.66		−0.07	− 23.58	− 0.086	−13.65
Child age in (months)	− 0.003	3.15		− 0.005	3.5	− 0.005	− 0.007
Child sex	0.0002	−0.04		−0.002	− 0.23	− 0.002	−0.4
Mother education (years)	−0.18	9.51		0.19	21.97	0.21	36.63
Wealth index	0.27	93.3		0.27	88.86	0.28	71.48
Household size	−0.04	−2.02		−0.004	− 0.13	− 0.08	−5.42
Skilled birth attendant	0.16	22.7		0.009	−0.25	0.18	17.55
Place of delivery	0.06	−3.5		0.07	22.76	0.07	16.2
Residuals	−0.1781	−20.44		−0.476	−12.9	−0.663	− 22.38
Total	0.0193	100		−0.018	100	−0.096	100

% represents the percentage contribution of each determinant’s CIX to the overall CIX

TDHS represents Tanzania Demographic and Health Surveys

### Decomposition of change in socioeconomic inequality in child

We revealed that inequalities in stunting among children aged 3–59 months didn’t change significantly. Due to the observed insignificant changes of inequalities in stunting, we will not get useful information by decomposing the observed changes in the concentration index (Table 2).

## Discussion

This study aimed at determining the trend, contributing factors, and changes of inequalities in stunting among children aged 3–59 months from 2004/5 to 2015/16. Over the years, both stunting and socioeconomic inequalities in stunting declined although burdening among the poor. Socioeconomic inequalities in stunting were mostly accounted for by the differentials in wealth index although the contribution declined over time. This was followed by the differentials in maternal years of schooling whose contribution increased over time. The differences in rural and urban areas reduced socioeconomic inequalities in stunting, the contribution declined over time.

The observed declining trend of stunting in Tanzania may be explained by increased agricultural production of maize, wheat, groundnuts and soghum [36] which are important for child nutrition and growth. Comparing to other nations in Africa and the world as whole, agricultural production in Tanzania is supported by favourable climatic condition and fertile soil [36]; political commitment through establishment of agricultural development agenda including the National Strategy for Growth and Reduction of Poverty 2005/6–2009/10 (MKUKUTA I) and 2010/11–2014/15(MKUKUTA II); and the Tanzania Five-Year DevelopmentPlan 2011/12–2015/16 [37] and the Kilimo Kwanza [38]. Furthermore, preasence of peace and security has been an asset for good nutrition status comparing to Eritrea and Burundi having a prevalence stunting of 50.3 and 57.5% respectively [39] which have been facing political instabilities and hence refugees thereby lacking food to feed lactating mothers and young children and hence impoper political commitment in agricultural and food production activities.

The observed insignificant decline in socioeconomic inequalities in stunting may be attributed by the role of Tanzania Social Action Fund (TASAF) which empowered communities in accessing, requesting and implementing projects towards improved livelihoods of the poor [40]; Big Results Now (BRN) enhanced availability of health services at ward levels for example dispensaries, availability of primary and secondary schools at ward levels [14, 40, 41] which increased school enrollment. Other scholars found an increasing trend of socioeconomic inequalities in stunting [9, 31, 42]. This may be accounted for by the methodological differences in estimating the levels of inequalities in stunting namely generalized CIX while other researchers used the Wagstaff [43] or Erreygers [44] CIX. Increased efforts by the government and other stakeholders are needed inorder to strengthen economic empowerment through entrepreneurship skills and education on child care practices among women of reproductive age.

Most of the determinants except the area of residence had a positive impact on socioeconomic inequalities in stunting. This is explained by the higher risk of stunting among the disadvantaged socioeconomic groups who were majority thus the combined marginal effect of each determinant influenced socioeconomic status. The larger contribution of the disparities in household wealth index on the socioeconomic inequalities in stunting may be explained by the majority of stunted children living in poor and poorest wealth indexes. Similar results by [26, 27] implyd that equal distribution of stunting across socio-economic groups will be attained if efforts are embarked on equal distribution of socio services like roads, water, and hospitals which will easy transportation of raw materials for construction of houses, availability of safe water at the household level and health monitoring respectively.

The increased contribution of differentials in maternal years of schooling on inequalities in stunting may be explained by a larger number of mothers with lower levels of education who have a small number of years in schools and had a large number of stunted children. Women from economically disadvantaged households are either not getting more opportunities to achieve better education or after being enrolled they drop out. These findings were consistent with [26, 27]. These findings may imply strengthening of education among women of reproductive age principally through adult education, continued programs under Big Results Now (BRN), increasing number of female enrollment in schools as well as mass media exposure and nutrition campaign.

The declined contribution of differentials in area of residence on the socioeconomic inequalities in stunting may be explained by governmental efforts of distributing socioeconomic infrastructures in both rural and urban areas which bridges up the gap. These results were consistent with other scholars [30, 45], although the area of residence may be interlinked with other socioeconomic factors including wealth and level of education [29]. Furthermore, the effect of area of residence on stunting was confounded by wealth index.

We didn’t decompose the changes in concentration indexes to get the factors for the changes in inequalities in stunting as there were no significant changes in CIXs between any two surveyed. We did not access scholars who found insignificant changes in inequalities in stunting, although our findings can be compared to [9] who found insignificant changes in inequalities in wasting when decomposing the changes in inequalities in malnutrition and hence didn’t decompose the CIX for wasting. Insignificant changes in inequalities in stunting means that disparities in stunting didn’t significantly increase or decrease. A big lesson is that, as we have identified the determinants of unequal distribution of stunting in each surveyed year, tackling these determinants may bring a significant decrease in the unequal distribution of stunting which is the required step towards attaining zero stunting and under-nutrition at large.

This paper contributes to the existing literature on different aspects. Firstly, we provide country estimates of the current levels of stunting among children aged 3–59 months where stunting is accurately captured rather than involving even children aged below 3 months. Although the minor difference in the proportions of stunted children was observed as compared to [18] for the survey year 2015/6. Secondly, we assessed how socioeconomic inequalities in stunting evolve, thus to our best knowledge this is the first study to examine the trend in socioeconomic inequalities in stunting to Tanzania using the most three recent DHS. Thirdly, had a larger sample size which gives enough power to make conclusions on the socioeconomic determinants of inequalities in stunting across surveyed years. Last but not least the study puts more emphasis on optimal health growth, as we evaluated child health equity using Wagstaff and Watanabe decomposition methods of the CIX.

Despite the drawn conclusions basing on our study findings, these results should be interpreted with caution as the study faced several limitations. Firstly, inadequate information collected from the respondent for-instance lack of information on religion and maternal feeding practices and dietary diversity; lacking these information give the room of worrying about confounding effects as these variables were not included in the model. Secondly, children not living with their mothers were not included in this study; this may lead to biased estimates. Thirdly, the comparability problem of the household wealth index across surveyed years because different items were used in the construction of wealth index across surveyed years. Fifth, the study adhered to Wagstaff and Wantanabe decomposition methods which are limited to continuous variables or dichotomous variables. Last but not least, the study investigated stunting without taking into account of nutritional diseases including underweight and wasting which may be interlinked with stunting.

## Conclusion

To attain a significant reduction of stunting among the disadvantaged groups and hence zero stunting by 2030, policies should encompass economic empowering of the socio-disadvantaged group for-instance; through housing, employment, and income. Secondly, the continued provision of free education specifically among the socio-disadvantaged groups thereby capacitating graduates through employment is very vital. Thirdly, further researches should incorporate maternal feeding practices and food taboos as they affect fetal growth during pregnancy. Last but not least, studies on socioeconomic inequalities in wasting and underweight are vital as they are interlinked with stunting.

## Data Availability

The datasets were downloaded from DHS measure website via http://dhsprogram.com/data/available-datasets.cfm

## Abbreviations

ANC: Antenatal care
BRN: Big Results Now
BMI: Body Mass Index
CIX: Concentration index
DHS: Demographic and Health Surveys
HAZ: Height for Age Z- scores
MDGs: Millennium Development Goals
MKUKUTA: Mkakati wa Kukuza na Kupunguza Umaskini Tanzania
SDGs: Sustainable Development Goals
svy: Survey
TDHS: Tanzania Demographic and Health Surveys
TASAF: Tanzania Social Action Fund

## References

[1] Unicef. Levels and Trends in Child Malnutrition: UNICEF / WHO / World Bank Group Joint Child Malnutrition Estimates. Midwifery. 2018;12. https://www.unicef.org/media/60626/file/Joint-malnutrition-estimates-2019.pdf

[2] Hoddinott J, Alderman H, Behrman JR, Haddad L, Horton S (2013). The economic rationale for investing in stunting reduction. Matern Child Nutr.

[3] Koncz V, Geldsetzer P, Manne-Goehler J, Wendt AS, Teufel F, Subramanian SV (2019). Shorter height is associated with diabetes in women but not in men: nationally representative evidence from Namibia. Obesity..

[4] Santos CDDL, Clemente APG, Martins VJB, Albuquerque MP, Sawaya AL. Adolescents with mild stunting show alterations in glucose and insulin metabolism. J Nutr Metab. 2010;2010:1–4. https://www.ncbi.nlm.nih.gov/pmc/articles/PMC3034971/pdf/JNUME2010-943070.pdf 10.1155/2010/943070 PMC3034971 21318152

[5] Rolfe EDL, De França GVA, Vianna CA, Gigante DP, Miranda JJ, Yudkin JS (2018). Associations of stunting in early childhood with cardiometabolic risk factors in adulthood. PLoS One.

[6] Olofin I, McDonald CM, Ezzati M, Flaxman S, Black RE, Fawzi WW, et al. Associations of Suboptimal Growth with All-Cause and Cause-Specific Mortality in Children under Five Years: A Pooled Analysis of Ten Prospective Studies. PLoS One. 2013;8(5):1–8. 10.1371/journal.pone.0064636 PMC3667136 23734210

[7] Hoffman DJ, Sawaya AL, Verreschi I, Tucker KL, Roberts SB (2000). Why are nutritionally stunted children at increased risk of obesity? Studies of metabolic rate and fat oxidation in shantytown children from Sao Paulo. Brazil Am J Clin Nutr.

[8] Deborah A, Kavita S, Elisabeth S, Lesley O, Tara K, Joyceline K, et al. Reducing malnutrition in Tanzania:estimates to support nutrition advocacy: Tanzania PROFILES 2014 [internet]. Reducing Malnutrition in Tanzania: Estimates to Support Nutrition Advocacy Tanzania PROFILES. 2014;2014 Available from: https://www.fantaproject.org/sites/default/files/resources/Tanzania-PROFILES-Report-2014-June2017.pdf.

[9] Kien VD, Lee H-Y, Nam Y-S, Oh J, Giang KB, Van MH. Trends in socioeconomic inequalities in child malnutrition in Vietnam: findings from the Multiple Indicator Cluster Surveys, 2000–2011. 2016;9716:1–8. https://www.tandfonline.com/doi/pdf/10.3402/gha.v9.29263?needAccess=true 10.3402/gha.v9.29263 PMC4780091 26950558

[10] Hangoma P, Aakvik A, Robberstad B (2017). Explaining changes in child health inequality in the run up to the 2015 millennium development goals (MDGs): the case of Zambia. PLoS One.

[11] Kröger H, Pakpahan E, Hoffmann R. What causes health inequality? A systematic review on the relative importance of social causation and health selection. Eur J Pub Health. 2015;25(6):951–60. 10.1093/eurpub/ckv111 26089181

[12] UNICEF, WHO, Word Bank Group. Levels and trends in child malnutrition. Geneva: Unicef; 2019. https://www.unicef.org/media/60626/file/Joint-malnutrition-estimates-2019.pdf.

[13] Crochemore I, Silva M (2018). da, França G V, Barros AJ, Amouzou a, Krasevec J, et al. socioeconomic inequalities persist despite declining stunting prevalence in low- and middle-income countries. J Nutr.

[14] MoHCDGEC, NBS, ICF (2016). Demographic and health survey and malaria Indicator survey 2015–16.

[15] IRS (2017). Overcoming the Challenges of Under-Nutrition in Tanzania Through 2021: Reflecting on Trends, Questions, and Future Scenarios for Urgent Nutrition Action. [Internet]. Inter-Agency Regional Analysts Network (IRAN).

[16] Mgongo M, Chotta NAS, Hashim TH, Uriyo JG, Damian DJ, Stray-pedersen B (2017). Underweight , Stunting and Wasting among Children in Kilimanjaro Region , Tanzania; a Population-Based Cross-Sectional Study.

[17] United Republic of Tanzania (2018). Tanzania National Nutrition Survey.

[18] Chirande L, Charwe D, Mbwana H, Victor R, Kimboka S, Issaka AI (2015). Determinants of stunting and severe stunting among under-fives in Tanzania: evidence from the 2010 cross-sectional household survey. BMC Pediatr.

[19] Khamis AG, Mwanri AW, Ntwenya JE, Kreppel K (2019). The influence of dietary diversity on the nutritional status of children between 6 and 23 months of age in Tanzania. BMC Pediatr.

[20] Rutstein SO, Rojas G (2006). Guide to DHS statistics [internet].

[21] De Onis M, Blössner M (1997). WHO Global Database on Child Growth and Malnutrition. Programme of Nutrition World Health Organization Geneva.

[22] Rutstein SO, Johnson K (2018). DHS Comparative Reports No.6: The DHS Wealth Index. MEASURE DHS.

[23] Rutstein SO, Rojas G (2006). Guide to DHS Statistics. Demographic and Health Surveys Methodology [Internet]. DHS Publication.

[24] Ruel MT, Alderman H (2013). Nutrition-sensitive interventions and programmes: how can they help to accelerate progress in improving maternal and child nutrition?. Lancet.

[25] Bhutta ZA, Das JK, Rizvi A, Gaffey MF, Walker N, Horton S (2013). Evidence-based interventions for improvement of maternal and child nutrition: what can be done and at what cost?. Lancet..

[26] Van De Poel E, Speybroeck N (2014). Decomposing malnutrition inequalities between scheduled castes and tribes and the remaining Indian population. Ethn Health.

[27] Rabbani A, Khan A, Yusuf S, Adams A. Trends and determinants of inequities in childhood stunting in Bangladesh from 1996 / 7 to 2014. Int J Equity Health. 2016:1–14. Available from:. 10.1186/s12939-016-0477-7. 10.1186/s12939-016-0477-7 PMC5112749 27852266

[28] Modern G, Sauli E, Mpolya E (2020). Correlates of diarrhea and stunting among under-five children in Ruvuma, Tanzania; a hospital-based cross-sectional study. Sci African.

[29] Paraje G (2009). Child stunting and socio-economic inequality. CEPAL Rev.

[30] Zere E, McIntyre D (2003). Inequities in under-five child malnutrition in South Africa. Int J Equity Health.

[31] Akombi BJ, Agho KE, Renzaho AM, Hall JJ, Merom DR (2019). Trends in socioeconomic inequalities in child undernutrition: evidence from Nigeria demographic and health survey (2003 – 2013). PLoS One.

[32] Donnell OO, Wagstaff A, Lindelow M (2008). Analyzing health equity using household survey data [internet].

[33] Kakwani N, Wagstaff A, Van DE (1997). Socioeconomic inequalities in health : Measurement , computation , and statistical inference.

[34] Rutstein SO, Kiersten K (2004). DHS comparative report 6: the DHS wealth index.

[35] Kakwani N, Wagstaff A, van Doorslaer E. Socioeconomic inequalities in health: Measurement, computation, and statistical inference. J Econom. 1997;77.

[36] Leliveld A, Dietz T, Foeken D, Klaver W (2013). Agricultural dynamics and food security trends in Uganda. Developmental Regimes in Africa (DRA) Project.

[37] United Republic of Tanzania (2016). Agricultural Sector Development Strategy-IIA 2015/2016–2024/2025. United Repub Tanzania [Internet].

[38] Mbunda R (2011). Kilimo Kwanza and Small Scale Producers : an Opportunity or a Curse?.

[39] WHO. Nutrition in the WHO Afrcican region. 7. 2557.

[40] TASAF. Tanzania Second Social Action Fund: First Quarter Implementation Progress Report (July–September 2011) [Internet]. 2011. Available from: www.tasaf.org:

[41] Ministry of Health. The National Road Map Strategic Plan to Improve Reproductive, Maternal, Newborn, Child and Adolescent Health in Tanzania - One Plan II; 2016. p. 142. https://www.globalfinancingfacility.org/sites/gff_new/files/Tanzania_One_Plan_II.pdf.

[42] Salvucci V. Determinants and trends of socioeconomic inequality in child malnutrition: the case of Mozambique, 1996-2011. Annu Conf Hum Dev Capab Assoc New Delhi 2008;168(10–13):1–30. https://onlinelibrary.wiley.com/doi/epdf/10.1002/jid.3135.

[43] Wagstaff A, Watanabe N (2000). Socioeconomic Inequalities in Child Malnutrition in the Developing World. The Development Dilemma.

[44] Erreygers G. Beyond the Health Concentration Index: An Atkinson Alternative for the Measurement of the Socioeconomic Inequality of Health. Biol Res. 2005;37.

[45] Angdembe MR, Dulal BP, Bhattarai K, Karn S. Increased risk of low birthweight and preterm birth, stunting in infancy, short adult height, poor schooling, and higher adult fasting glucose concentrations. The. Int J equity health [internet]. 2019;18(42):1–17. Available from. 10.1186/s12939-019-0944-z. 10.1186/s12939-019-0944-z PMC6402091 30836975

